# TRIM37 Augments AP-2γ Transcriptional Activity and Cellular Localization via K63-linked Ubiquitination to Drive Breast Cancer Progression

**DOI:** 10.7150/ijbs.69466

**Published:** 2022-07-04

**Authors:** Guimei Cui, Zhuoran Gao, Shiehong Chang, Nitin Narwade, Yitian Chen, Barun Poudel, Kate M. K. Lei, Weibo Zhang, Gang Li, Terence C. W. Poon, Edwin Cheung

**Affiliations:** 1 Cancer Centre, Faculty of Health Sciences, University of Macau, Macau SAR, China.; 2 Centre for Precision Medicine Research and Training, Faculty of Health Sciences, University of Macau, Macau SAR, China.; 3 MoE Frontiers Science Center for Precision Oncology, University of Macau, Macau SAR, China.; 4 Pilot Laboratory, University of Macau, Macau SAR, China.; 5 Institute of Translational Medicine, University of Macau, Macau SAR, China.; 6 Faculty of Health Sciences, University of Macau, Taipa, Macau SAR, China.

**Keywords:** AP-2γ, TRIM37, breast cancer, transcriptional regulation

## Abstract

Activator Protein 2 gamma (AP-2γ) is a master transcription factor that plays a critical role in the development and progression of breast cancer. However, the underlying mechanism is still unclear. Herein, using a proteomics approach, we identified Tripartite motif-containing 37 (TRIM37) as a novel coactivator of AP-2γ-mediated transcription in breast cancer cells. We demonstrate that TRIM37 facilitates AP-2γ chromatin binding to directly regulate the AP-2γ mediated transcriptional program. We also show that TRIM37 achieves this by stimulating K63 chain-linked ubiquitination of AP-2γ, promoting protein localization from the cytoplasm to the nucleus. In clinical analyses, we find TRIM37 is upregulated in multiple breast cancer datasets, supporting our findings that the TRIM37-AP-2γ interaction is essential for breast cancer tumor growth. Overall, our work reveals that TRIM37 is an oncogenic coactivator of AP-2γ in breast cancer and provides a novel therapeutic target for treating the disease.

## Introduction

Breast cancer (BCa) is the most commonly diagnosed cancer in women 1. Many determinants control the development and progression of the disease, including transcription factors such as Activator protein-2 gamma (AP-2γ)/Transcription Factor Activator Protein-2 gamma (TFAP2C) 2. The TFAP2C gene is located on chromosome 20q13 and is frequently amplified in BCa 3, 4, but whether its expression is associated with overall survival remains unclear 5-7. However, elevated AP-2γ expression is correlated with a decreased response to endocrine therapy 8-10. As a DNA binding transcription factor, AP-2γ binds to cis-regulatory elements in the genome called AP-2γ binding sites (AP2GBS) and recruits coregulatory factors, such as ERα, GATA3, PELP1, and PRC1 PRC2 9, 11-14. Together, these factors function in an organized manner to facilitate the transcription of essential genes required for oncogenesis and progression of BCa 2, 15.

Tripartite motif-containing 37 (TRIM37), another critical regulator of BCa 14, is a member of the TRIM superfamily of proteins, characterized by an RBCC motif consisting of a RING finger, two B-boxes, and a coiled-coil domain 16. TRIM37 is located on chromosome 17q23, in a region amplified in many cancers, including about 40% of BCa 14, 17. A high TRIM37 protein expression is associated with a poor prognosis in BCa 14, consistent with the role of TRIM37 in promoting BCa cell proliferation, migration, and invasion 14, 18-20. Mechanistically, TRIM37 has been shown to function as a transcriptional corepressor, silencing the expression of tumor suppressor genes by mono-ubiquitinating histone H2A 14.

The transcriptional network controlling BCa is highly complex and tightly controlled, involving the coordinated efforts of many DNA binding transcription factors, cofactors, and target genes 11, 21, 22. While numerous studies indicate TRIM37 and AP-2γ as critical regulators in BCa, it is unclear if there is any functional interplay between these two factors. Herein, using RIME (Rapid Immunoprecipitation Mass Spectrometry of Endogenous Proteins) 23, we identified TRIM37 as an AP-2γ-associated protein. We also demonstrate that TRIM37 is a coactivator of AP-2γ-mediated transcription. Finally, our findings suggest that TRIM37 regulates transcriptional activity by acting as an E3-ligase mediating K63-linked ubiquitination of AP-2γ, promoting the translocation of the protein from the cytoplasm to the nucleus.

## Results

### TRIM37 interacts with AP-2γ in BCa cells

**Figure 1:**
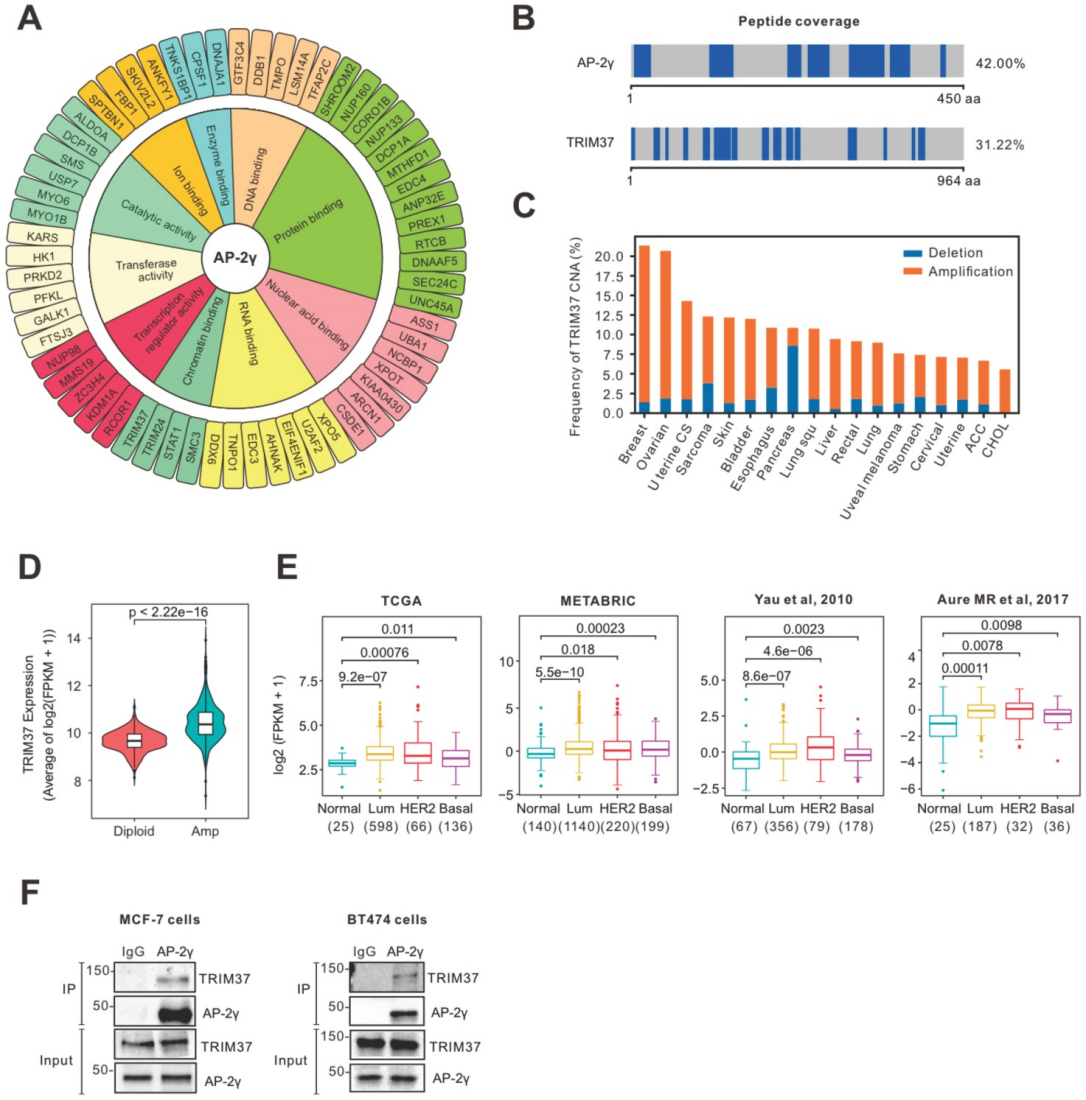
** RIME analysis reveals AP-2γ is associated with TRIM37 in BCa cells. A,** Graphical plot showing AP-2γ-associated proteins in MCF-7 cells. The clustering of the graph is based on molecular functions. **B,** Peptide coverage, number of unique peptides (highlighted in blue) identified in AP-2γ and TRIM37 from the AP-2γ RIME analysis. **C,** TRIM37 mRNA alteration in different cancer types from TCGA. **D,** TRIM37 expression in TRIM37 diploid versus TRIM37-amplified tumors (TCGA). **E,** TRIM37 mRNA levels in normal and different BCa subtypes from different publicly available datasets. The number of patients and p-values are shown as indicated. **F,** Validation of the AP-2γ-TRIM37 interaction by co-immunoprecipitation. Nuclear AP-2γ protein in MCF-7 (left) or BT474 (right) cells was immunoprecipitated, and TRIM37 was detected by immunoblotting. IgG was used as a negative control.

To identify cooperating factors involved in regulating AP-2γ-mediated transcription, we isolated endogenous proteins associated with AP-2γ in MCF-7 cells using RIME analysis. We considered proteins are associated with AP-2γ if they meet the following criteria: a) the associated protein is present in all three independent replicates, b) the associated protein shows a minimum of five-fold change in intensity ratio compared to the IgG control, and c) the number of unique peptides representing the associated protein is more than four. With these criteria, we identified 89 high-confident AP-2γ-associated proteins (Supplementary Table 1). A Gene Ontology (GO) analysis of these AP-2γ-associated proteins shows they are involved in diverse molecular functions, such as protein binding, DNA binding, RNA binding, and catalytic activity (Fig. 1A). We also ranked these proteins based on their enrichment scores as a general indicator of interaction strength (Fig. S1). Among this list of proteins, TRIM37 was one of the strongest and most confident AP-2γ protein interactors (Fig. 1B).

TRIM37 is an essential factor in BCa 14, 24, 25. In agreement with previous findings 17, we observed TRIM37 amplified in many cancers, including BCa (Fig. 1C). We also found that TRIM37 amplification in BCa is associated with increased mRNA expression (Fig. 1D) and, in general, expressed higher in luminal and HER2 than basal BCa subtypes (Fig. 1E). To explore the potential interplay between TRIM37 and AP-2γ in BCa, we validated the TRIM37-AP-2γ interaction by performing independent co-immunoprecipitation analysis in MCF-7 and BT474 cells. As shown in Fig. 1F, the TRIM37-AP-2γ interaction is present in both cell lines. Together, our results suggest that TRIM37 is a novel AP-2γ-associated protein highly expressed in BCa.

### TRIM37 is colocalized with AP-2γ on chromatin

**Figure 2:**
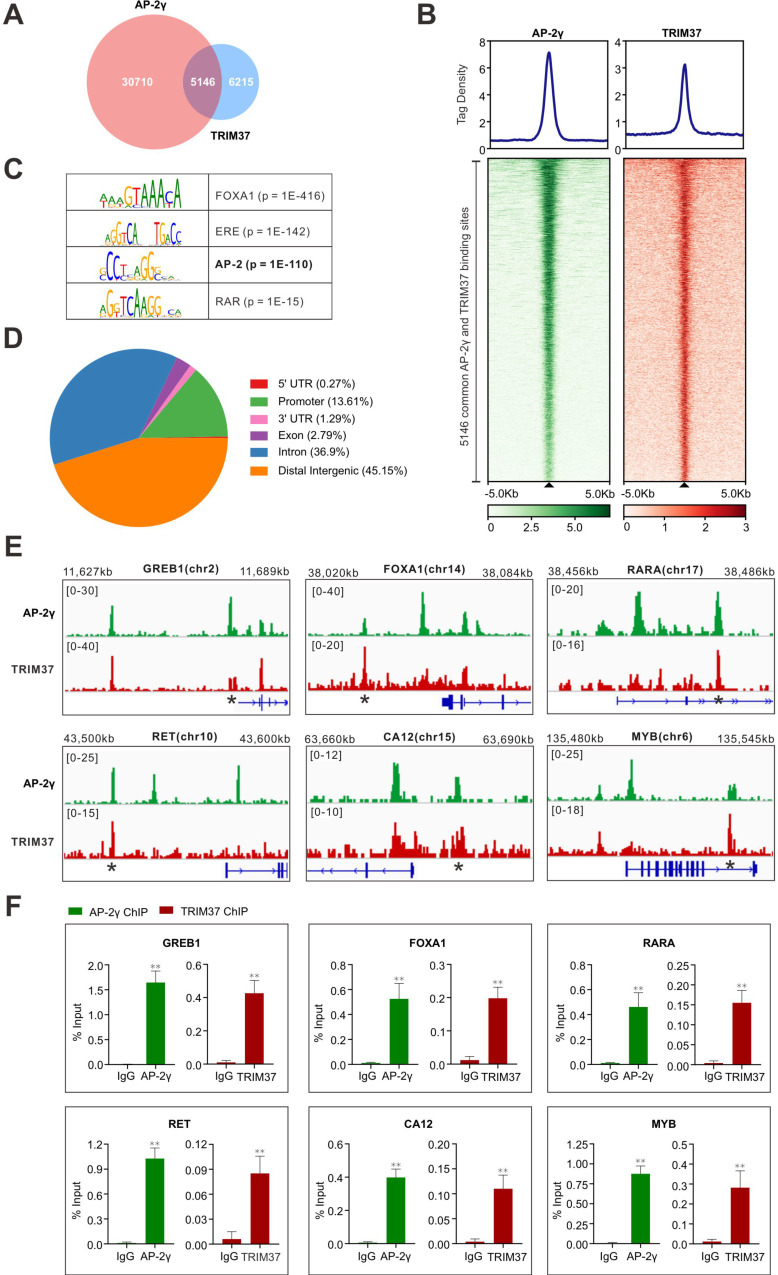
** TRIM37 binding is co-localized with AP-2γ. A,** Venn diagram showing the overlap of AP-2γ and TRIM37 ChIP-seq peaks. **B,** Graphs showing the average tag density for AP-2γ and TRIM37 at AP-2γ-TRIM37 co-binding regions (top). Heatmaps showing the binding intensity signals for the 5,146 sites ranked from the strongest to the weakest binding sites (below). **C,** Motif enrichment analysis of TRIM37 binding sites. Significant top enriched motifs in TRIM37 ChIP-seq peaks are shown with corresponding p-values. **D,** Pie chart illustrating the genomic distribution of TRIM37 binding regions relative to the whole genome. **E,** Genomic snapshots of AP-2γ and TRIM37 ChIP-seq peaks at the regulatory regions of model genes. Asterisks denote binding sites used for validation in F. **F,** ChIP-qPCR validation of AP-2γ and TRIM37 co-occupancy at model genes in MCF-7 cells. Data are represented as a percentage of input chromatin immunoprecipitated. Error bars show ± SD from at least 3 independent experiments. * *P* < 0.05; ** *P* < 0.01.

To establish whether TRIM37 plays a role in regulating AP-2γ dependent transcription in BCa, we began by asking whether TRIM37 and AP-2γ binding is colocalized on chromatin. For this, we performed TRIM37 and AP-2γ ChIP-seq in MCF-7 cells. Overall, we identified 11,361 TRIM37 binding sites (T37BS) and 35,856 AP-2γ binding sites (AP2GBS) (Fig. 2A and B). A motif enrichment analysis of T37BS revealed an enrichment of the AP-2 binding motif (Fig. 2C), suggesting TRIM37 is recruited to chromatin via AP-2γ. When we overlapped the binding sites for TRIM37 and AP-2γ, we found that 45.3% (5,146) of T37BS are shared with AP2GBS (Fig. 2A), further supporting the RIME findings that TRIM37 interacts with AP-2γ. A genomic distribution analysis of T37BS showed that TRIM37 is mainly occupied at distal intergenic (45.15%), intron (36.90%), and gene promoter regions (13.61%) (Fig. 2D). Next, we searched whether the two factors are co-bound near known AP-2γ regulated genes 2, 9, 26. As shown in Fig. 2E, TRIM37 and AP-2γ co-binding sites are significantly enriched at the regulatory regions of GREB1, FOXA1, RARA, RET, CA12, and MYB. We validated these co-binding events independently by ChIP-qPCR (Fig. 2F). Together, these results suggest AP-2γ regulates transcription by recruiting TRIM37 to chromatin.

### TRIM37 is required for efficient AP-2γ dependent transcription

**Figure 3:**
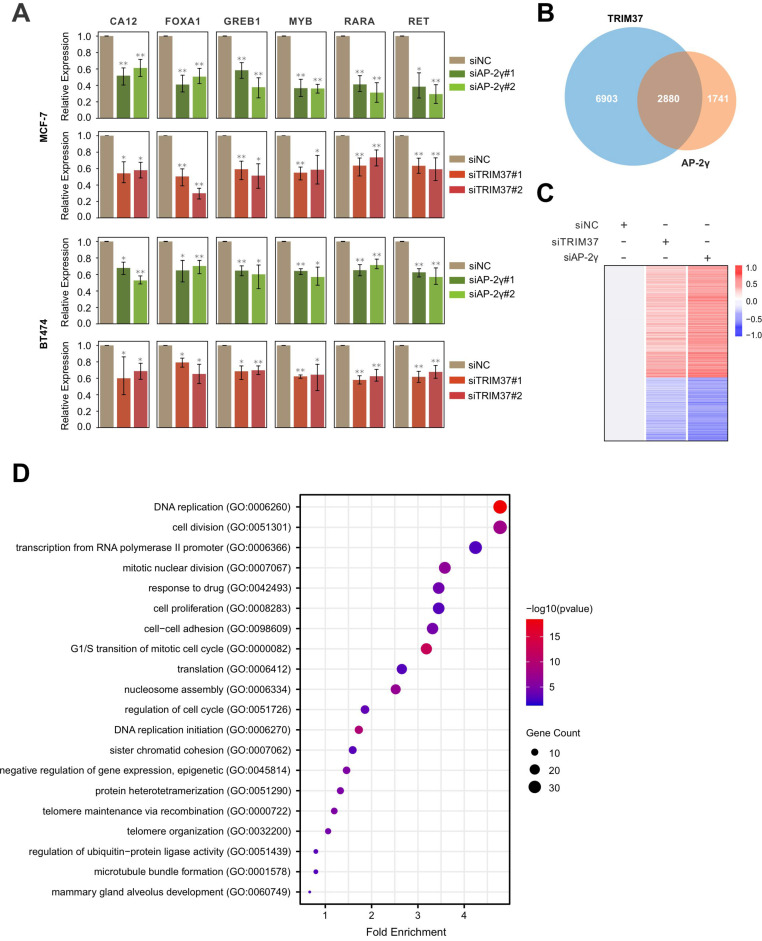
** TRIM37 is required for efficient AP-2γ dependent transcription. A,** Effect of AP-2γ and TRIM37 knockdown on the expression of AP-2γ-regulated genes. MCF-7 cells (upper panel) and BT474 cells (lower panel) were transfected with siAP-2γ, siTRIM37, or siNC. Gene expression was measured by RT-qPCR and normalized against GAPDH. Error bars show ± SD from at least 3 independent experiments. * *P* < 0.05; ** *P* < 0.01. **B,** Venn diagram showing AP-2γ and TRIM37 coregulated genes. MCF-7 cells treated with siNC, siAP-2γ, or siTRIM37 were profiled by RNA-seq. A total of 2,880 genes are coregulated by AP-2γ and TRIM37 (AP-2γ FC > 1.5, TRIM37 FC > 1.2). **C,** Heatmap showing RNA-seq gene expression analysis of MCF-7 cells treated with siAP-2γ, siTRIM37, or siNC. A total of 968 downregulated genes (blue) and 1,581 upregulated genes (red) (AP-2γ FC > 1.5, TRIM37 FC > 1.2) were identified. **D,** GO analysis of downregulated genes (coactivated by TRIM37 and AP-2γ) (AP-2γ FC > 1.5, TRIM37 FC > 1.2).

Next, we determined if TRIM37 is essential for AP-2γ-mediated transcription. To do this, we examined the effect of siRNA-mediated silencing of TRIM37 on the expression of AP-2γ-regulated genes in MCF-7 and BT474 cells. Overall, depleting TRIM37 reduced the expression of AP-2γ-regulated genes in both cell lines (Fig. 3A). We also explored the requirement of TRIM37 on the global AP-2γ transcriptional program by performing RNA-seq on MCF-7 cells silenced with siTRIM37 or siAP-2γ. From our analysis, we identified 2,880 TRIM37 and AP-2γ coregulated genes (Fig. 3B). Among these coregulated genes, 968 are downregulated, and 1,581 are upregulated after TRIM37 and AP-2γ depletion (Fig. 3C). This result suggests that TRIM37 can function as a coactivator and a corepressor of transcription. For this work, we focused on the coactivator activity of TRIM37 in AP-2γ-mediated transcription. GO term analysis of TRIM37 and AP-2γ coactivated genes shows they are enriched for biological processes associated with DNA replication, cell cycle, cell division, and cell proliferation (Fig. 3D). Together, our results demonstrate that TRIM37 is required for efficient transcription of AP-2γ target genes and coregulate cancer-associated genes that affect tumor progression.

### TRIM37 enhances AP-2γ binding to chromatin

**Figure 4:**
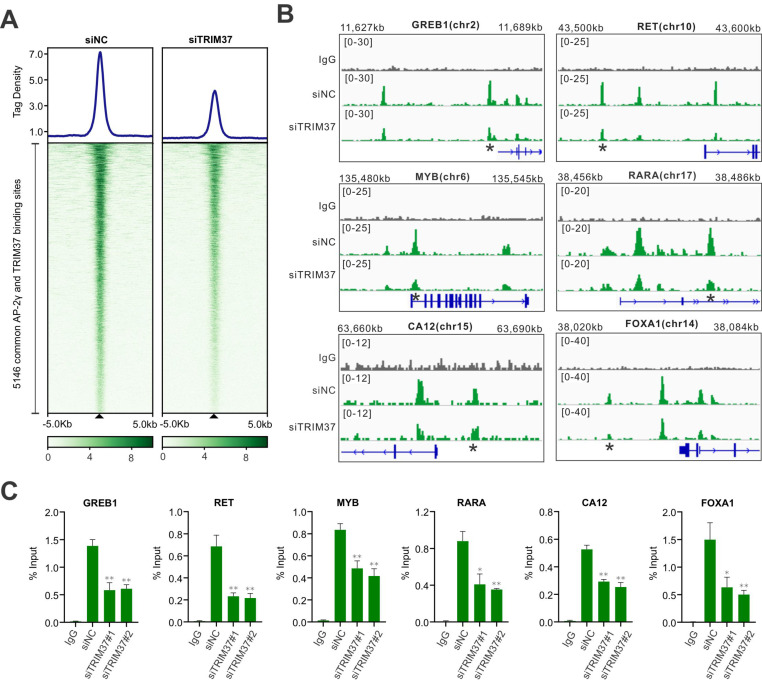
** TRIM37 affects AP-2γ binding to chromatin. A,** Graph showing the average tag density (top) and heatmap (bottom) of AP-2γ at AP-2γ and TRIM37 co-bound regions with and without siTRIM37 treatment. **B,** Genomic snapshots of AP-2γ ChIP-seq peaks surrounding model genes with and without siTRIM37 treatment. **C,** ChIP-qPCR validation of TRIM37 knockdown on AP-2γ binding sites at model genes in MCF-7 cells. Data are represented as a percentage of input chromatin immunoprecipitated. Error bars show ± SD from at least 3 independent experiments. * *P* < 0.05; ** *P* < 0.01.

To understand how TRIM37 stimulates AP-2γ mediated transcription, we assessed whether TRIM37 could be involved in modulating AP-2γ binding to chromatin. We tested this possibility by performing an AP-2γ ChIP-seq on MCF-7 cells before and after TRIM37 depletion. As shown in Fig. 4A, depleting TRIM37 significantly reduced the average binging intensity of AP-2γ at the 5,146 TRIM37 and AP-2γ co-binding sites, including binding sites associated with AP-2γ-regulated genes (Fig. 4B and C). Together, our results suggest that TRIM37 promotes or stabilizes AP-2γ binding to chromatin.

### TRIM37 promotes AP-2γ nuclear translocation via K63 ubiquitination

**Figure 5:**
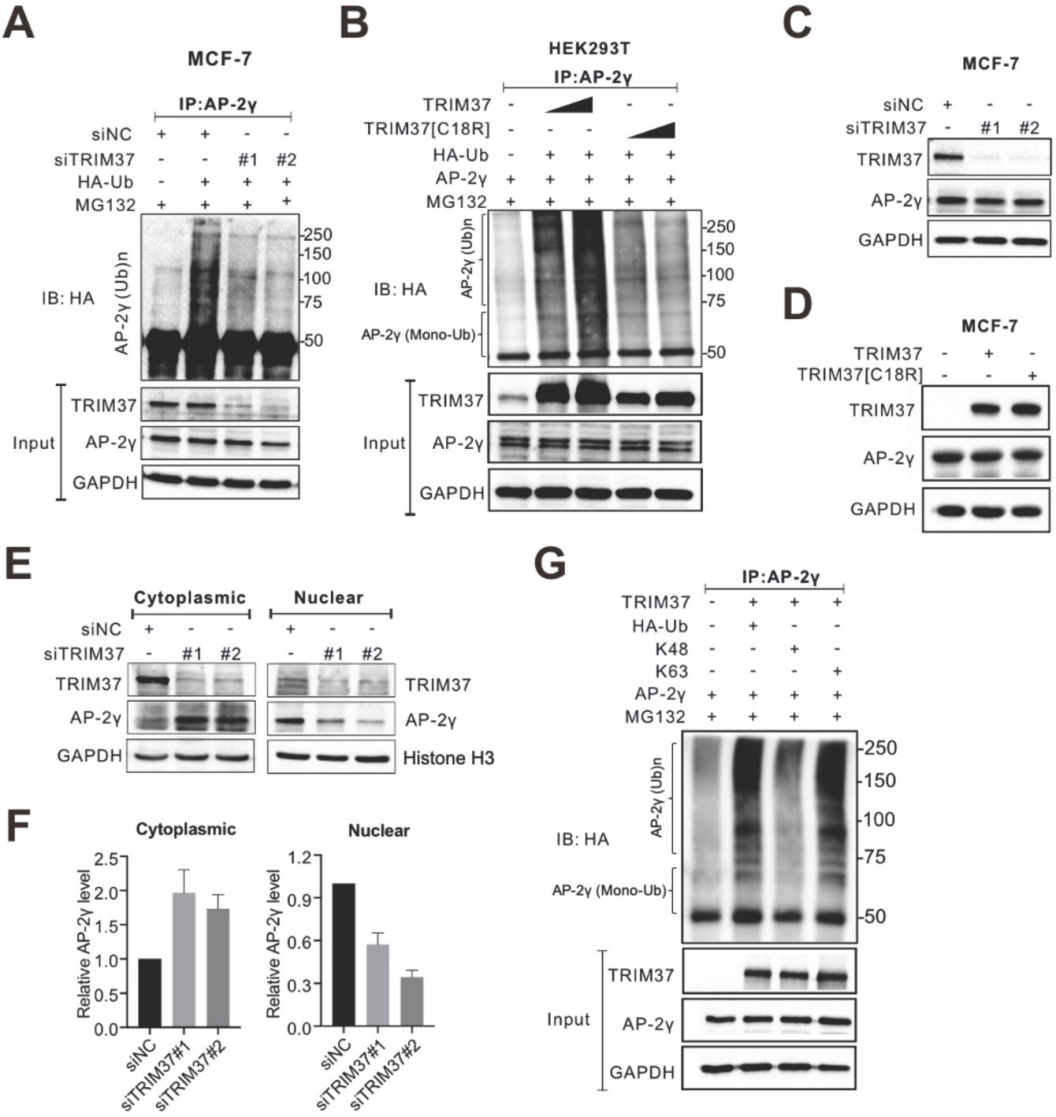
** TRIM37 affects AP-2γ nuclear translocation via K63 ubiquitination. A,** MCF-7 cells transfected with siNC or siTRIM37 were treated with MG132 before lysis and then subjected to anti-AP-2γ IP followed by immunoblot analysis with anti-HA. **B,** HEK293T cells were transfected with constructs expressing AP-2γ and wild-type TRIM37 or TRIM37[C18R] mutant as indicated. Samples were processed as described in A. The asterisks indicate the mono-ubiquitinated forms of AP-2γ. **C,** MCF-7 cells were transfected with siNC or siTRIM37. Cell lysis was collected at 72 h post-transfection and subjected to immunoblot analysis using the indicated antibodies. **D,** MCF-7 cells were transfected with constructs expressing TRIM37 or TRIM37[C18R]. Samples were processed as described in C. **E,** Cell fractions were prepared from MCF-7 cells treated with siNC or siTRIM37. GAPDH and histone H3 served as loading controls and cell fraction markers. **F,** Quantitation of cytoplasmic (left) or nuclear (right) AP-2γ normalized to the loading control. Error bars show ± SD from at least 3 independent experiments. * *P* < 0.05; ** *P* < 0.01. **G,** *In vivo* ubiquitination assays were performed in HEK293T cells. Cells were transiently transfected with HA-tagged K63-only ubiquitin or K48-only ubiquitin. The asterisks indicate the mono-ubiquitinated forms of AP-2γ.

TRIM37 is an E3 ubiquitin ligase 14, 25, 27, 28. Thus, we speculated that TRIM37 might function as a coactivator by ubiquitinating AP-2γ. Indeed, siRNA-mediated depletion of TRIM37 decreased AP-2γ ubiquitination levels in MCF-7 cells (Fig. 5A). Consistent with these results, overexpression of TRIM37 in HEK 293T cells increased AP-2γ ubiquitination levels in a dose-dependent manner (Fig. 5B). Our results also show that TRIM37 can induce mono- and polyubiquitination of AP-2γ (Fig. 5B and Fig. S3). Moreover, a RING domain mutant of TRIM37 (C18R, which destroyed the E3 ubiquitin ligase activity) substantially reduced the ability of TRIM37 to promote the ubiquitination of AP-2γ (Fig. 5B), further supporting our idea that TRIM37 is an E3 ubiquitin ligase for AP-2γ.

Next, we asked whether TRIM37 regulates AP-2γ protein expression levels. Overall, the depletion of TRIM37 did not change AP-2γ protein levels in MCF-7 (Fig. 5C) or BT474 cells (Fig. S2A). Consistent with these observations, the overexpression of TRIM37 also did not affect AP-2γ protein levels (Fig. 5D). Since TRIM37 does not modulate AP-2γ protein expression levels in BCa cells, we decided to see if TRIM37 could alter AP-2γ subcellular localization instead. Fractionation analyses show that AP-2γ protein level decreased in the nucleus and increased in the cytoplasm after TRIM37 knockdown in MCF-7 (Fig. 5E and F) and BT474 cells (Fig. S2B and S2C). These observations suggest that TRIM37 affects AP-2γ protein translocation rather than protein stability. Lysine-48 (K48)-linked ubiquitin chains mainly target proteins for proteasomal degradation, whereas K63-linked ubiquitin chains mediate nonproteolytic signals, including those regulating subcellular localization, protein activation, and protein-protein interaction 29-31. To determine which ubiquitin chain TRIM37 uses to modify AP-2γ, we performed ubiquitination assays containing either K63-only or K48-only ubiquitin as substrates. Our results show that TRIM37 promotes K63 but not K48 ubiquitination of AP-2γ (Fig. 5G). Taken together, our results indicate that TRIM37 functions as a coactivator of AP-2γ-mediated transcription by promoting the translocation of the protein from the cytoplasm to the nucleus through K63-mediated ubiquitination of AP-2γ.

### TRIM37 is essential for BCa growth

**Figure 6:**
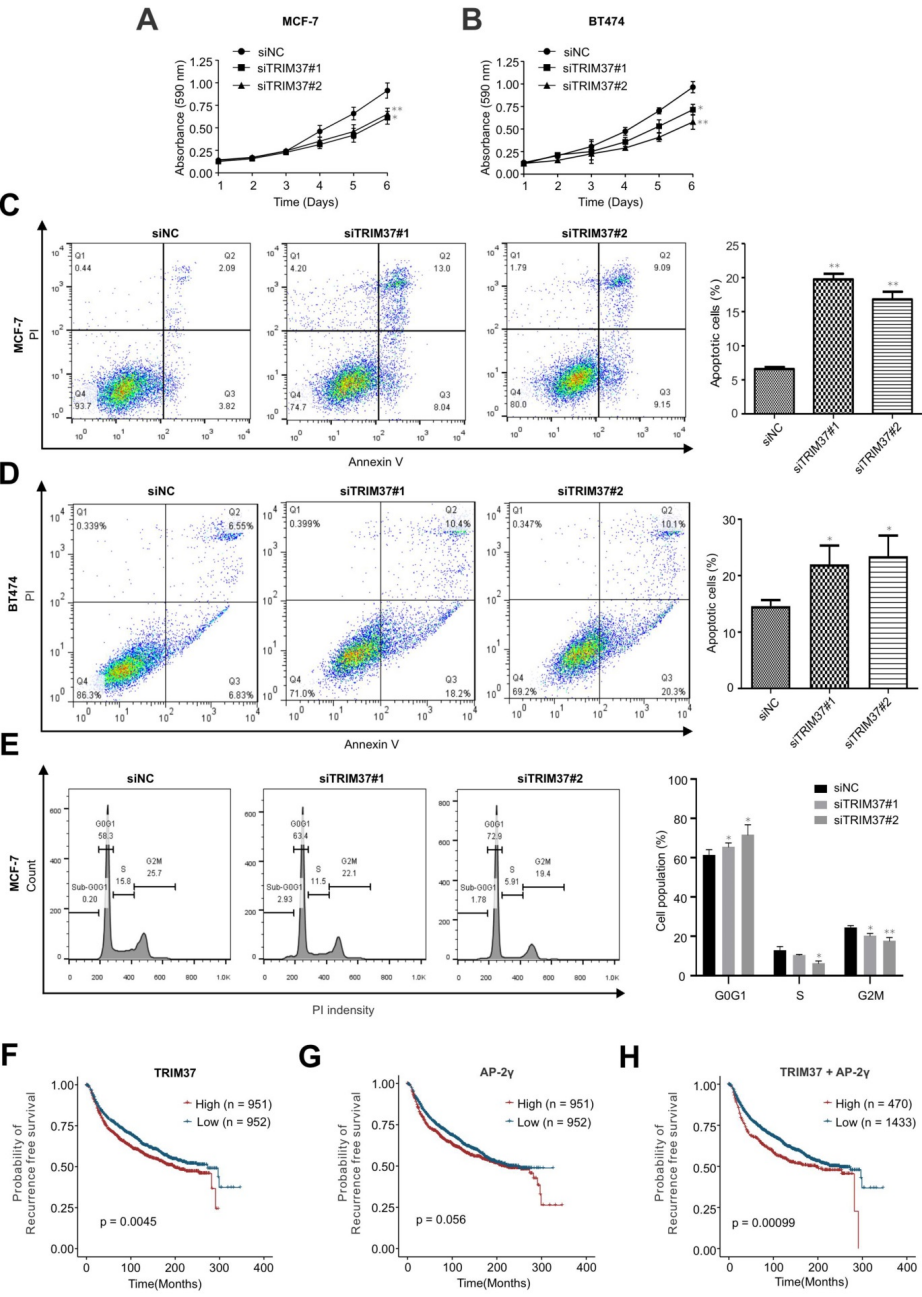
** TRIM37 is essential for BCa growth. A and B,** MTT assays were performed on (A) MCF-7 and (B) BT474 cells transfected with siNC or siTRIM37. Error bars represent ± SD from 3 independent experiments. * *P* < 0.05; ** *P* < 0.01. **C and D,** Cell apoptosis analyses by flow cytometry of (C) MCF-7 and (D) BT474 transfected with siNC or siTRIM37. Error bars show ± SD from at least 3 independent experiments. * *P* < 0.05; ** *P* < 0.01. **E,** Cell cycle analysis of MCF-7 cells transfected with siNC or siTRIM37 were detected by flow cytometry. Error bars show ± SD from at least 3 independent experiments. * *P* < 0.05; ** *P* < 0.01. **F-H,** Kaplan-Meier plots showing the recurrence-free survival of BCa patients for TRIM37 (F), AP-2γ (G), TRIM37, and AP-2γ (H) expression in the METABRIC dataset. BCa patients were stratified into high (red) and low (blue) groups based on the median expression of the target gene. Significance was calculated using a log-rank test (*P* < 0.05).

In addition to examining the role of TRIM37 in AP-2γ-mediated transcription, we also addressed its biological functions in BCa. To do this, we depleted TRIM37 in MCF-7 and BT474 cells and assayed for cell proliferation (MTT) and apoptosis. Knockdown of TRIM37 markedly reduced cell proliferation (Fig. 6A and B) and significantly increased cell apoptosis (Fig. 6C and D). We also examined the effect of TRIM37 on the cell cycle. FACs analyses show that depletion of TRIM37 increased the G1/S transition and decreased the S phase of the mitotic cell cycle (Fig. 6E). Together, our results suggest TRIM37 is essential for BCa proliferation and survival through promoting the cell cycle and inhibiting apoptosis.

### TRIM37 and AP-2γ expression is a good predictor of BCa survival

Finally, since the above findings indicate TRIM37 and AP-2γ function together, we were interested in determining whether these two factors have good prognostic value in predicting BCa survival. Our analysis found that AP-2γ expression is not associated with BCa patient survival (Fig. 6G). In contrast, high TRIM37 expression is significantly associated with a poor survival outcome (Fig. 6F). Although TRIM37 alone can predict survival, we explored whether we could obtain a better prognostic indicator if we considered TRIM37 and AP-2γ expression together. Indeed, our results show that the separation and significance in BCa survival increased if we used the expression information of both factors (Fig. 6H). Taken together, our clinical analysis suggests combining TRIM37 and AP-2γ expression for predicting BCa patient outcomes.

## Discussion

AP-2γ is a critical player in the development and progression of BCa 9, 10. Furthermore, transcriptional regulation by AP-2γ is influenced by a specific combination of coregulatory factors 32-34. Thus, knowing all the interactors of AP-2γ and understanding their function is essential for potential BCa therapy. Over the years, through various biochemical and genomic approaches, only several AP-2γ coregulatory proteins have been identified thus far, including ERα, FOXA1, FBP1, KDM5B, MYC, MTA1, B23, and CITED 2, 11, 35-39. In this study, using a highly robust proteomics approach, we identified all chromatin-bound AP-2γ interacting proteins in BCa cells (Fig. 1). We also revealed TRIM37 as a novel coregulator of AP-2γ and provided functional insight into how TRIM37 regulates AP-2γ transcriptional activity in BCa cells.

TRIM37 is an oncogenic transcriptional regulator in BCa, monoubiquitinating histone H2A and suppressing the transcription of a cohort of tumor suppressor genes 14. The findings from our work also support TRIM37 as an oncoprotein. We showed that TRIM37 is highly expressed in BCa and is required for promoting BCa cell growth (Fig. 1E and 6A-B). Our findings also show that TRIM37 is a component of the AP-2γ transcriptional complex essential for AP-2γ-mediated transcription (Fig. 2 and 4). In addition, our work reveals that TRIM37 functions as a coactivator of AP-2γ regulated gene transcription (Fig. 3) by enhancing AP-2γ chromatin binding (Fig. 4).

In addition to monoubiquitinating histone H2A, TRIM37 has also been reported to activate the NF-kB pathway via polyubiquitinating TRAF2 and promote peroxisomal matrix protein import by monoubiquitinating PEX5 14, 18, 27. In this study, we identified a new function for TRIM37. We show that TRIM37 interacts with AP-2γ and mediates K63-mediated mono- and polyubiquitination of AP-2γ, promoting AP-2γ nuclear translocation (Fig. 5 and S3). We also showed that the RING domain of TRIM37 is required for AP-2γ ubiquitination but is not necessary for the interaction between TRIM37 with AP-2γ (data not shown), suggesting that TRIM37 may interact with AP-2γ by recruiting other protein factors. This possibility and the specific interaction site between TRIM37 and AP-2γ will be tested in future work.

Our global gene expression analysis reveals that TRIM37 regulates the transcription of pro-mitotic genes (Fig. 3). In support of this observation, cell proliferation and apoptosis results show that TRIM37 promotes cell proliferation and inhibits apoptosis (Fig. 6A-D). Notably, two recent studies reported that centrosome depletion induces synthetic lethality in TRIM37 amplified cancer cells 24, 25. Specifically, when PLK4 is inhibited, TRIM37 degrades CEP192, a core component of the pericentriolar centrosome material. This degradation leads to the unsuccessful assembly of the mitotic spindle resulting in the failure of mitosis and cell proliferation. Together, these findings suggest that TRIM37 is a promising therapeutic target for BCa treatment.

Finally, our analysis of clinic data shows that high expression of TRIM37 in BCa patients is associated with a poor survival outcome (Fig. 6F). Furthermore, we found that combining AP-2γ expression information with TRIM37 data provides a better predictive value than TRIM37 only. Taken together, the results from this study suggest that TRIM37 and AP-2γ expression can help better predict BCa patient outcomes.

In conclusion, we identified TRIM37 as a novel interactor of AP-2γ, regulating AP-2γ-mediated transcription via K63-linked ubiquitination. The resulting posttranslational modification of AP-2γ moves the protein from the cytoplasm to the nucleus. This study also provides novel insights into the transcriptional network and the molecular mechanisms by which AP-2γ regulates gene expression in BCa. Finally, our work will directly lead to significant advances in understanding the role of TRIM37-AP-2γ interaction in gene transcription and aid in developing novel therapeutics for BCa treatment.

## Materials and Methods

### Antibodies and reagents

The following antibodies were used for RIME, ChIP, Co-IP and immunoblotting: anti-AP-2γ (sc-12762x, SCBT), anti-AP-2γ (sc-8977, SCBT), anti-TRIM37 (A301-174A, Bethyl Lab), anti-HA (sc-7392, SCBT), normal mouse IgG (sc-2025, SCBT), normal rabbit IgG (sc-2027, SCBT), and anti-GAPDH (sc-47724, SCBT). MG132 (s2619) was purchased from Selleckchem. Dynabeads^TM^ M-280 sheep anti-mouse IgG (11202D) and Dynabeads^TM^ protein G (10009D) were purchased from Invitrogen.

### Plasmids

The plasmid vectors, p3X-Flag-Myc-CMV-26 and p3X-Flag-Myc-CMV-26-TRIM37, were generous gifts from Michael R. Green (University of Massachusetts Medical School). The TRIM37[C18R] mutant construct was generated using the QuickChange site-directed mutagenesis kit (Agilent). The primer used for the mutagenesis is listed in Supplementary Table 2. The full-length TFAP2C cDNA was cloned into the pCMV5 vector at KpnI and HindIII. The pRK5-HA and pRK5-HA-K48/K63 ubiquitin plasmids were purchased from Addgene.

### Cell culture

MCF-7 cells (ATCC) were cultured in DMEM (Gibco) supplemented with 5% FBS (Gibco). HEK293T and BT474 cells (ATCC) were cultured in DMEM with 10% FBS. All cells were grown at 37 °C in a 5% CO_2_ incubator.

### RNAi studies

Cells were seeded in a six-well plate or 10 cm dish to 70% confluency and subsequently transfected with 5 nM of Dicer-substrate short interfering RNA (IDT) using Lipofectamine RNAiMAX (Invitrogen). Then, 72 h after transfection, cells were harvested for RT-qPCR, RNA-seq, western blotting, or ChIP assay. Two separate siRNAs were used for every target and paired with negative control, siNC. siRNA and RT-qPCR primers sequences are listed in Supplementary Table 2.

### Cell proliferation assays

Cells were transfected with siNC or siTRIM37, seeded into 96-well plates at 3 × 10^3^ cells/well, and then incubated with 5 mg/mL sterilized MTT (Life Technologies) in PBS for 3 h at 37 °C. The MTT was removed, and 100 µl DMSO was added (Sigma). The plate was then incubated for a further 30 min at 37 °C. The absorbance was measured at an OD of 590 nm using a plate reader (Thermo Scientific). Cell proliferation was monitored every day for 6 days. Experiments were performed in 3 technical replicates and repeated 3 independent times.

### Cell apoptosis assays

Cell apoptosis assay was performed using the Dead Cell Apoptosis Kit (Invitrogen) according to the manufacturer's instructions. In brief, MCF-7 cells were transfected with siNC or siTRIM37. After 72 h of transfection, cells were harvested, centrifuged at 1,200 rpm for 5 min at 4 °C, washed with cold PBS once, and then resuspended in a binding buffer containing Annexin V-FITC and propidium iodide. Next, cells were incubated for 15 min in the dark at room temperature. After staining, the cells were mixed with 400 µl binding buffer and immediately analyzed by flow cytometry (BD FACS Calibur). Experiments were repeated three independent times.

### *In vivo* ubiquitination assays

For ubiquitination assays with MCF-7, cells were first transfected with siNC or siTRIM37 for 24 h after transfection. Then, the media was changed, and cells were transfected with the HA-ubiquitin construct. Finally, cells were treated with 20 nM MG132 for 6 h before harvesting. As for ubiquitination assays with HEK293T, cells were transfected with the HA-ubiquitin plasmid together with other indicated constructs. Cells were then treated with MG132 under the same condition as MCF-7 cells. After 48 h of transfection, cells were harvested and lysed with RIPA buffer containing NEM (Sigma) and protein inhibitor cocktail (Roche). Ubiquitinated proteins were precipitated with anti-AP-2γ pre-bound to sheep anti-mouse magnetic beads and analyzed by WB.

### Co-immunoprecipitation assay

Cells were grown in 15 cm dishes. Cell lysates (same as the ChIP method) were incubated with 5 µg of AP-2γ antibody and 100 µl magnetic beads complex on a rotator overnight at 4 °C. Afterward, the protein-antibody-beads complex was washed with RIPA lysis buffer, resuspended in sample buffer, boiled for 10 min, and analyzed by WB.

### Subcellular fractionation

For subcellular fractionation analysis, cytoplasmic and nuclear proteins were separated using the subcellular protein fractionation kit according to the manufacturer (Abmon). Briefly, cells were harvested as a pellet and were incubated with fractionation buffer at 4 °C for 5 min. Next, the lysate was centrifuged 500 × g at 4 °C for 5 min. The supernatant (cytoplasmic extract) was transferred to a clean tube, while the pellet was sequentially added with disruption buffer and incubated at 4 °C for 10 min. Finally, the supernatant (nuclear protein) was collected, and protein sample buffer was added and boiled for 6 min. The sample was then analyzed by WB.

### RIME analysis

AP-2γ RIME was performed as described 23 with minor modifications. Briefly, 1 × 10^7^ MCF-7 cells were crosslinked with 1% mass spectrometry grade formaldehyde (Pierce, Cat # 28906) for 8 min at RT. 10 µg anti-AP-2γ (sc12762x) antibody pre-bound with 100 µl sheep anti-mouse Dynabeads was used for immunoprecipitation. Immunoprecipitated proteins were extracted by heating in denaturing buffer (0.02 M Tris/HCl, pH 6.9, 2% SDS, 0.1 M dithiothreitol) for 10 minutes. After spinning at 14,000 × g for 30 sec, the supernatant containing the extracted proteins was collected and stored at -80 °C until proteomic analysis. To predict the GO categories of AP-2γ-associated proteins, we used msarc 1.4.5 (https://CRAN.R-project.org/package=msarc).

### Label-free LC-MS/MS quantitative proteomic analysis

The extracted proteins were subjected to trypsin digestion using the filter-aided sample preparation approach described by Wiśniewski JR, 2018 40. The tryptic digest was cleaned up with C18 ZipTip (Millipore) according to the manufacturer's instructions. After drying down with a speedvac, the cleaned peptides were reconstituted in water with 5% acetonitrile (can) and 0.1% formic acid. Peptides were separated on an EASY-Spray™ LC Column 75 µm × 50 cm, 2-µm 100-Å pore size (Thermo Fisher Scientific) with an ACN gradient from 5% to 35% in 0.1% formic acid over 65 min. Peptides eluting from the column were analyzed on a Q Exactive mass spectrometer (Thermo Fisher Scientific) operated in a data-dependent mode. Survey full-scan MS spectra (400-2,500 m/z) were acquired at 70,000 FWHM resolution. The top 20 most intense ions were sequentially isolated and fragmented in the orbitrap by higher-energy collisional dissociation (HCD) with normalized collision energy (NCE) set to 25. MS/MS spectra were acquired at 17,500 FWHM resolution. The LC-MSMS data were searched against the UniProt human protein sequence (reviewed) database using MaxQuant v1.5.3.17 41 to identify proteins with a minimum of 2 unique peptides at PSM FDR of 0.01, peptide FDR of 0.01, and protein FDR of 0.01. For label-free quantification, the LFQ intensity of each unique protein was calculated using the intensity data of the unmodified unique peptides and razor peptides.

### Chromatin Immunoprecipitation (ChIP)

The TRIM37 ChIP was performed as described 42, except we used a two-step method to cross-link the cells 43. Briefly, MCF-7 cells were cultured in a 15 cm dish to approximately 1 × 10^7^ cells. Cells were then treated with the protein-protein cross-linker, DSG (Pierce), at a final concentration of 2 mM at room temperature for 45 min. After washing with PBS for 3×, cells were treated with 1% formaldehyde and incubated at RT for 10 min. Nuclear extracts made from these double cross-linked cells were then immunoprecipitated with anti-TRIM37 or normal rabbit antibody pre-bound to protein G Dynabeads (Invitrogen). The precipitated DNA was de-crosslinked at 65 °C for 8-18 h and subjected to either high-throughput sequencing or ChIP-qPCR. The primers used for amplifying ChIP products are listed in Supplementary Table 2.

### ChIP-seq analysis

ChIP-seq libraries were prepared using the TruSeq ChIP Library Prep kit (Illumina) and subjected to paired-end sequencing. Quality control and pre-processing of raw data were processed using the Fastp software (version 0.20.1). Reads were mapped to the hg19 genome (ftp://ftp.ccb.jhu.edu/pub/data/bowtie2_indexes/hg19.zip) with Bowtie 2 44 and duplicates were removed using the Picard v.2.20 tool 45. MACS14 (version 1.4.2) was used for peak calling (q value ≤ 1e-04) 46. The Chipseeker 47 package in R statistical environment (R x64 3.6.3) was used to annotate the genomic region of the peaks. The motif enrichment analysis was performed using homer HOMER v4.11.1 (http://homer.ucsd.edu/homer/motif/).

### RNA-seq

Raw data quality control and pre-processing were processed with Fastp (version 0.20.1) and Trim Galore (version 0.6.6). Clean reads were mapped to the hg19 genome with HISAT2 (version 2.2.1) 48. RSeQC (version 2.6.2) 49 was used to check mapped read quality. Transcripts Per Million (TPM) was counted using StringTie (version 2.1.4) (https://www.gencodegenes.org/human/release_19.html) 50. Differential gene expression analysis was performed based on TPM values with a cut-off of 1.5 for siAP-2γ/siNC and 1.2 for siTRIM37/siNC. We plotted the overlapping differentially expressed genes using the heatmap R package and performed GO enrichment analysis using DAVID (https://david.ncifcrf.gov/).

### Clinic data analysis

TCGA and Yau breast cancer datasets 51 were downloaded from UCSC Xena (https://xenabrowser.net/datapages/). The METABRIC breast cancer dataset was downloaded from cBioPortal (https://www.cbioportal.org/datasets). The Aure dataset 52 (GSE80999) was downloaded from GEO. TRIM37 expression values and clinical sample type information were extracted from these datasets. The student's t-test was used to calculate the *p*-value. We used Survival v3.2-11 and Survminer v0.4.9 for survival analysis. We used the log-rank test to compare the survival curves of the two groups obtained based on the median expression.

### Statistics

All RT-qPCR and ChIP results were collected from at least 3 biological replicates to achieve statistical significance. The difference between groups was determined using a two-tailed student *t*-test with Prism v7 (Graphpad). Significant differences were considered when *P* < 0.05; * *P* < 0.05 and ** *P* < 0.01.

### Data availability

All sequencing data from this work have been submitted to the NCBI Gene Expression Omnibus (GEO) under accession number GSE182547.

## Supplementary Material

Supplementary figures and tables.

## References

[1] Sung H, Ferlay J, Siegel RL, Laversanne M, Soerjomataram I, Jemal A (2021). Global Cancer Statistics 2020: GLOBOCAN Estimates of Incidence and Mortality Worldwide for 36 Cancers in 185 Countries. CA Cancer J Clin.

[2] Cyr AR, Kulak MV, Park JM, Bogachek MV, Spanheimer PM, Woodfield GW (2015). TFAP2C governs the luminal epithelial phenotype in mammary development and carcinogenesis. Oncogene.

[3] Kallioniemi A, Kallioniemi O-P, Piper J, Tanner M, Stokke T, Chen L (1994). Detection and mapping of amplified DNA sequences in breast cancer by comparative genomic hybridization. Proceedings of the National Academy of Sciences.

[4] Tanner MM, Tirkkonen M, Kallioniemi A, Holli K, Collins C, Kowbel D (1995). Amplification of chromosomal region 20q13 in invasive breast cancer: prognostic implications. Clinical Cancer Research.

[5] Pellikainen J, Kataja V, Ropponen K, Kellokoski J, Pietiläinen T, Böhm J (2002). Reduced nuclear expression of transcription factor AP-2 associates with aggressive breast cancer. Clinical Cancer Research.

[6] Perkins SM, Bales C, Vladislav T, Althouse S, Miller KD, Sandusky G (2015). TFAP2C expression in breast cancer: correlation with overall survival beyond 10 years of initial diagnosis. Breast cancer research and treatment.

[7] Zhao C, Yasui K, Lee CJ, Kurioka H, Hosokawa Y, Oka T (2003). Elevated expression levels of NCOA3, TOP1, and TFAP2C in breast tumors as predictors of poor prognosis. Cancer.

[8] Gee JMW, Eloranta J, Ibbitt J, Robertson J, Ellis I, Williams T (2009). Overexpression of TFAP2C in invasive breast cancer correlates with a poorer response to anti-hormone therapy and reduced patient survival. The Journal of Pathology: A Journal of the Pathological Society of Great Britain and Ireland.

[9] Woodfield GW, Chen Y, Bair TB, Domann FE, Weigel RJ (2010). Identification of primary gene targets of TFAP2C in hormone responsive breast carcinoma cells. Genes, Chromosomes and Cancer.

[10] Woodfield GW, Horan AD, Chen Y, Weigel RJ (2007). TFAP2C controls hormone response in breast cancer cells through multiple pathways of estrogen signaling. Cancer research.

[11] Tan SK, Lin ZH, Chang CW, Varang V, Chng KR, Pan YF (2011). AP-2γ regulates oestrogen receptor-mediated long-range chromatin interaction and gene transcription. The EMBO journal.

[12] Krendl C, Shaposhnikov D, Rishko V, Ori C, Ziegenhain C, Sass S (2017). GATA2/3-TFAP2A/C transcription factor network couples human pluripotent stem cell differentiation to trophectoderm with repression of pluripotency. Proceedings of the National Academy of Sciences.

[13] Liu J, Liu Z, Li M, Tang W, Pratap UP, Luo Y (2021). Interaction of transcription factor AP-2 gamma with proto-oncogene PELP1 promotes tumorigenesis by enhancing RET signaling. Molecular oncology.

[14] Bhatnagar S, Gazin C, Chamberlain L, Ou J, Zhu X, Tushir JS (2014). TRIM37 is a new histone H2A ubiquitin ligase and breast cancer oncoprotein. Nature.

[15] Park J, Wu T, Cyr A, Woodfield G, De Andrade J, Spanheimer P (2015). The role of Tcfap2c in tumorigenesis and cancer growth in an activated Neu model of mammary carcinogenesis. Oncogene.

[16] Meroni G, Diez-Roux G (2005). TRIM/RBCC, a novel class of 'single protein RING finger' E3 ubiquitin ligases. Bioessays.

[17] Sinclair CS, Rowley M, Naderi A, Couch FJ (2003). The 17q23 amplicon and breast cancer. Breast Cancer Res Treat.

[18] Li Y, Deng L, Zhao X, Li B, Ren D, Yu L (2018). Tripartite motif-containing 37 (TRIM37) promotes the aggressiveness of non-small-cell lung cancer cells by activating the NF-κB pathway. The Journal of pathology.

[19] Zhao P, Guan H-T, Dai Z-J, Ma Y-G, Liu X-X, Wang X-J (2017). Knockdown of tripartite motif-containing protein 37 (TRIM37) inhibits the proliferation and tumorigenesis in colorectal cancer cells. Oncology research.

[20] Tang S-l, Gao Y-l, Wen-Zhong H (2018). Knockdown of TRIM37 suppresses the proliferation, migration and invasion of glioma cells through the inactivation of PI3K/Akt signaling pathway. Biomedicine & Pharmacotherapy.

[22] Nolens G, Pignon J-C, Koopmansch B, Elmoualij B, Zorzi W, De Pauw E (2009). Ku proteins interact with activator protein-2 transcription factors and contribute to ERBB2 overexpression in breast cancer cell lines. Breast Cancer Research.

[23] Mohammed H, Taylor C, Brown GD, Papachristou EK, Carroll JS, D'Santos CS (2016). Rapid immunoprecipitation mass spectrometry of endogenous proteins (RIME) for analysis of chromatin complexes. Nat Protoc.

[24] Yeow ZY, Lambrus BG, Marlow R, Zhan KH, Durin M-A, Evans LT (2020). Targeting TRIM37-driven centrosome dysfunction in 17q23-amplified breast cancer. Nature.

[25] Meitinger F, Ohta M, Lee K-Y, Watanabe S, Davis RL, Anzola JV (2020). TRIM37 controls cancer-specific vulnerability to PLK4 inhibition. Nature.

[26] Franke CM, Gu VW, Grimm BG, Cassady VC, White JR, Weigel RJ (2020). TFAP2C regulates carbonic anhydrase XII in human breast cancer. Oncogene.

[27] Wang W, Xia Z-J, Farré J-C, Subramani S (2017). TRIM37, a novel E3 ligase for PEX5-mediated peroxisomal matrix protein import. Journal of Cell Biology.

[28] Chen S, He Z, Zhu C, Liu Y, Li L, Deng L (2020). TRIM37 Mediates Chemoresistance and Maintenance of Stemness in Pancreatic Cancer Cells via Ubiquitination of PTEN and Activation of the AKT-GSK-3β-β-Catenin Signaling Pathway. Frontiers in oncology.

[29] Swatek KN, Komander D (2016). Ubiquitin modifications. Cell Res.

[30] Grumati P, Dikic I (2018). Ubiquitin signaling and autophagy. J Biol Chem.

[31] Dikic I, Wakatsuki S, Walters KJ (2009). Ubiquitin-binding domains - from structures to functions. Nat Rev Mol Cell Biol.

[32] Braganca J, Eloranta JJ, Bamforth SD, Ibbitt JC, Hurst HC, Bhattacharya S (2003). Physical and functional interactions among AP-2 transcription factors, p300/CREB-binding protein, and CITED2. J Biol Chem.

[33] Yang X, Phillips DL, Ferguson AT, Nelson WG, Herman JG, Davidson NE (2001). Synergistic activation of functional estrogen receptor (ER)-alpha by DNA methyltransferase and histone deacetylase inhibition in human ER-alpha-negative breast cancer cells. Cancer Res.

[34] Wong PP, Miranda F, Chan KV, Berlato C, Hurst HC, Scibetta AG (2012). Histone demethylase KDM5B collaborates with TFAP2C and Myc to repress the cell cycle inhibitor p21(cip) (CDKN1A). Mol Cell Biol.

[35] Kang H-J, Lee M-H, Kang H-L, Kim S-H, Ahn J-R, Na H (2014). Differential regulation of estrogen receptor α expression in breast cancer cells by metastasis-associated protein 1. Cancer research.

[36] Wong P-P, Miranda F, Chan KV, Berlato C, Hurst HC, Scibetta AG (2012). Histone demethylase KDM5B collaborates with TFAP2C and Myc to repress the cell cycle inhibitor p21 cip (CDKN1A). Molecular and cellular biology.

[37] Bamforth SD, Bragança J, Eloranta JJ, Murdoch JN, Marques FI, Kranc KR (2001). Cardiac malformations, adrenal agenesis, neural crest defects and exencephaly in mice lacking Cited2, a new Tfap2 co-activator. Nature genetics.

[38] Bragança J, Swingler T, Marques FI, Jones T, Eloranta JJ, Hurst HC (2002). Human CREB-binding protein/p300-interacting transactivator with ED-rich tail (CITED) 4, a new member of the CITED family, functions as a co-activator for transcription factor AP-2. Journal of Biological Chemistry.

[39] Lin C-Y, Chao A, Wang T-H, Lee L-Y, Yang L-Y, Tsai C-L (2016). Nucleophosmin/B23 is a negative regulator of estrogen receptor α expression via AP2γ in endometrial cancer cells. Oncotarget.

[40] Wiśniewski JR (2018). Filter-aided sample preparation for proteome analysis. Microbial Proteomics: Springer.

[41] Cox J, Mann M (2008). MaxQuant enables high peptide identification rates, individualized ppb-range mass accuracies and proteome-wide protein quantification. Nature biotechnology.

[42] Holmes KA, Brown GD, Carroll JS (2016). Chromatin Immunoprecipitation-Sequencing (ChIP-seq) for Mapping of Estrogen Receptor-Chromatin Interactions in Breast Cancer. Methods Mol Biol.

[43] Tian B, Yang J, Brasier AR (2012). Two-step cross-linking for analysis of protein-chromatin interactions. Methods Mol Biol.

[44] Langmead B, Salzberg SL (2012). Fast gapped-read alignment with Bowtie 2. Nature methods.

[45] Ebbert MT, Wadsworth ME, Staley LA, Hoyt KL, Pickett B, Miller J (2016). Evaluating the necessity of PCR duplicate removal from next-generation sequencing data and a comparison of approaches. BMC bioinformatics.

[46] Zhang Y, Liu T, Meyer CA, Eeckhoute J, Johnson DS, Bernstein BE (2008). Model-based analysis of ChIP-Seq (MACS). Genome biology.

[47] Yu G, Wang L-G, He Q-Y (2015). ChIPseeker: an R/Bioconductor package for ChIP peak annotation, comparison and visualization. Bioinformatics.

[48] Kim D, Langmead B, Salzberg SL (2015). HISAT: a fast spliced aligner with low memory requirements. Nature methods.

[49] Wang L, Wang S, Li W (2012). RSeQC: quality control of RNA-seq experiments. Bioinformatics.

[50] Pertea M, Kim D, Pertea GM, Leek JT, Salzberg SL (2016). Transcript-level expression analysis of RNA-seq experiments with HISAT, StringTie and Ballgown. Nature protocols.

[51] Yau C, Esserman L, Moore DH, Waldman F, Sninsky J, Benz CC (2010). A multigene predictor of metastatic outcome in early stage hormone receptor-negative and triple-negative breast cancer. Breast cancer research.

[52] Aure MR, Vitelli V, Jernström S, Kumar S, Krohn M, Due EU (2017). Integrative clustering reveals a novel split in the luminal A subtype of breast cancer with impact on outcome. Breast Cancer Research.

