# Supplementary material for: TRIM37 Augments AP-2γ Transcriptional Activity and Cellular Localization via K63-linked Ubiquitination to Drive Breast Cancer Progression
Source: Int J Biol Sci. 2022 Jul 4;18(11):4316–28. doi: 10.7150/ijbs.69466 (PMC9295074; doi:10.7150/ijbs.69466)
Supplement: Supplementary file 1 — Supplementary figures and tables. [file ijbsv18p4316s1.pdf]

TFAP2C	CPSF6	CCT6A	XPOT
EDC4	SEC13	SMC3	GALK1
EDC3	CORO1B	FBP1	PRRC2A
PREX1	ANP32E	PPL	MYO1B
DCP1A	CLIC3	MMS19	SF3A3
NUP160	TNPO1	STAT1	KARS
DCP1B	FTSJ3	PAICS	RCOR1
PRKD2	SEC24C	EIF4ENIF1	RCC2
TRIM37	TCP1	UNC45A	GTF3C4
ZC3H4	MTHFD1	HEATR6	RTCB
CMBL	SEC31A	DDB1	SART3
SHROOM2	MOV10	DNAJA1	HK1
TNKS1BP1	ECM29	KDM1A	CSDE1
ALDOA	TMPO	UBA1	U2AF2
COPB2	DNAAF5	OGT	COPB1
ANKFY1	PFKL	SMS	FAM120A
ASS1	CTNND1	TRIM24	SKIV2L2
TBC1D10A	MYO6	USP7	SPTBN1
LSM14A	BAG3	PDCD6IP	AHNAK
SCRIB	COPA	CPSF1	DDX6
COG3	NUP133	NUP98	
NCBP1	XPO5	ACP1	
KIAA0430	ARCN1	SAMHD1	

Extended Data Table 1: List of AP-2γ-associated proteins identified by RIME, related to Figure 1.

	Gene	Primer (5’-3’)
siRNA	TRIM37#1_F	GAAGGAUACUUGAAUCCACAAAATG
	TRIM37#1_R	CAUUUUGUGGAUUCAAGUAUCCUUCAU
	TRIM37#2_F	CCAGCUACGAGAACUAGUAAAUUGT
	TRIM37#2_R	ACAAUUUACUAGUUCUCGUAGCUGGAG
	AP-2γ#1_F	GCCCUGAUUGUCAUAGACAAAUCCT
	AP-2γ#1_R	AGGAUUUGUCUAUGACAAUCAGGGCUU
	AP-2γ#2_F	GGAAGAUUCUAUUUAAUUGAAACTC
	AP-2γ#2_R	GAGUUUCAAUUAAAUAGAAUCUUCCAA
qRT-PCR	TRIM37_F	CAGCCCCCGGATGAAGATAC
	TRIM37_R	CCATCAGGTGCAGTGTCAGT
	AP-2γ_F	GAAGAGGACTGCGAGGATCG
	AP-2γ_R	GCTGATATTCGGCGACTCCA
	GREB1_F	CAGGCTTTTGCACCGAATCT
	GREB1_R	CAAAGCGTGTCGTCTTCAGCT
	MYB_F	CGCAGCCATTCAGAGACACT
	MYB_R	GCTGCATGTGTGGTTCTGTG
	FOXA1_F	GGCTTCCTCTTCGCCCG
	FOXA1_R	AGTAGGCCTCCTGCGTGT
	RARA_F	GGGGGAATCCTGAATCGAGC
	RARA_R	CCATGTCCTGTGATGCTGCT
	CA12_F	CACACACATGGACGACCCTT
	CA12_R	CGCAGTACAGACTTGCACTTG
	RET_F	GAGCCCTCCCTTCCACATG
	RET_R	GGACTCTCTCCAGGCCAGTTC
	GAPDH_F	GGCCTCCAAGGAGTAAGACC
	GAPDH_R	AGGGGAGATTCAGTGTGGTG
ChIP	GREB1_F	GCCAGGGAGAAAGAGTTCCC
	GREB1_R	GGCAAATGCCACCGTTTC
	MYB_F	GGCAGAAATTCACGTGGCTG
	MYB_R	ACTGACTGAGCTGACCTCCA
	FOXA1_F	TCCCTTGCTCCACCCTTCTA
	FOXA1_R	ACTCAGGAAAGCAAGGACTGG
	RARA_F	GTACCCCGCAGGCAGTGT
	RARA_R	GGATAAAGCCACTCCAAGGTAGGT

	CA12_F	GGAGGAAAGAATGCCAGGCT
	CA12_R	GAAGCAGAAACACGGCAAGG
	RET_F	CTGAGTCAGACCTGCTGCC
	RET_R	CTGAGGTCCAGGGGCAAAAA
	MYB_KD_F	CTTCTCGCACCCTCCTTCAG
	MYB_KD_R	TGCTTTGCCTCCATGGACTT
Vector construction	TRIM37[C18R]_F	CATACGTATGAAACATCGGAAAACCTCAGC
	TRIM37[C18R]_R	CATACGTATGGAGAAATTGCGGGATG

Extended Data Table 2: List of primers used for quantitative real-time RT-PCR, ChIP, and plasmid construction.

Protein	Average intensity ratio
TFAP2C	3567.9
EDC4	3336.2
EDC3	2191.1
PREX1	1624.3
DCP1B	1142.1
NUP160	1099.0
DCP1A	1046.8
PRKD2	989.5
TRIM37	877.2
ZC3H4	796.4

Figure S1. List of top 10 AP-2γ-associated proteins identified by RIME. All the AP-2γ-associated proteins identified by RIME were ordered by the average intensity ratio from three independent experiments.

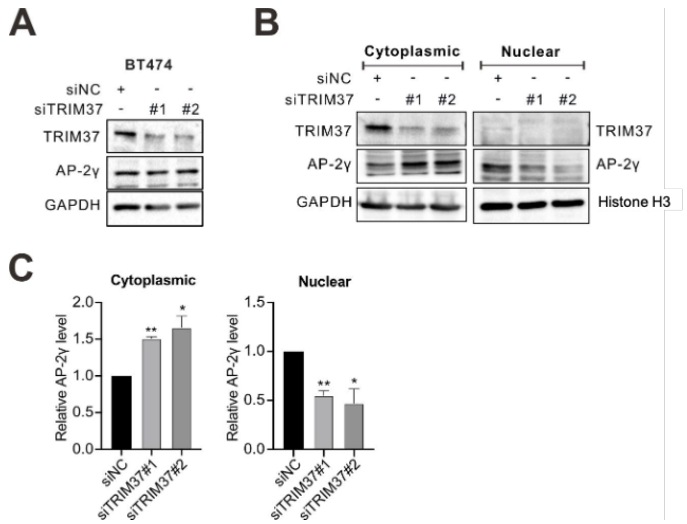

Figure S2. TRIM37 affects AP-2γ nuclear translocation in BT474 cells.

A, BT474 cells were transfected with siNC or siTRIM37. Cell lysis was collected at 72 h post-transfection and subjected to immunoblot analysis using the indicated antibodies.

B, Cell fractions were prepared from BT474 cells treated with siNC or siTRIM37. GAPDH and Histone H3 served as loading controls and cell fraction markers.

C, Quantitation of cytoplasmic (left) or nuclear (right) AP-2γ normalized to the loading control. Error bars show ± SD from 2 independent experiments. * $P < 0.05$; ** $P < 0.01$.

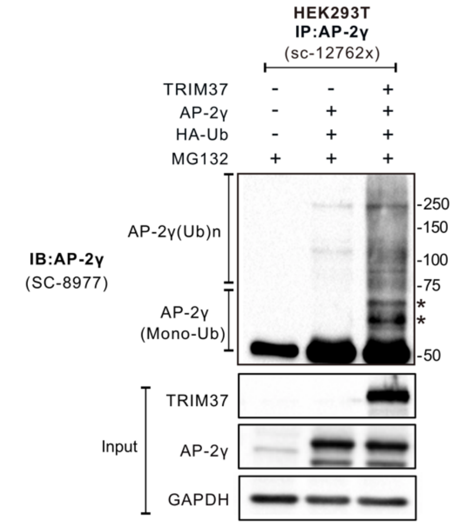

Figure S3. TRIM37 induces mono- and polyubiquitination of AP-2γ.

In vivo ubiquitination assays were performed in HEK293T cells by transiently transfecting the indicated constructs. Cell lysates were immunoprecipitated for AP-2γ followed by detection with the indicated antibodies. The asterisks indicate the mono-ubiquitinated forms of AP-2γ.
